# Role of Reorientation Training in Enhancement of the Knowledge Regarding Growth Monitoring Activities by Anganwadi Workers in Urban Slums of Delhi

**DOI:** 10.4103/0970-0218.39244

**Published:** 2008-01

**Authors:** Anuradha Davey, Sanjeev Davey, Utsuk Datta

**Affiliations:** Research Consultant HS-PROD, NIMS, ICMR, New Delhi, India; 1 SMO NPSP, WHO, New Delhi, India; 2 Department of Education and Training and MCHA, NIHFW, New Delhi, India

## Introduction

Early childhood developments constitute the foundation of the human development. Early years of the life are the most crucial period for the physical, mental, social, emotional, language development and lifelong learning. In a malnourished child, development of the milestones is delayed. Developmental delays are mainly observed in the areas like vision and fine motors, language and comprehension and personal social development. The delay was noticed to the extent of 7-11 months in these areas in different age groups.(1)

In urban areas, nutritional status of the slum children is poorer than their counterparts in the rural areas.(2) Two-third of the preschool children in the urban slum are underweight. According to NFHS-2 of Delhi, 35% of children under 3 years of age are underweight and 37% are stunted.(3) Anaemia is the most frequent malnutrition among the children from the slum community.(4)

ICDS scheme, in such scenario of health, occupies a significant place as an intervention in the socially and economically disadvantaged class of the society. The effective outcome of the nutrition services rendered through the Anganwadi centers (AWCs) depends on the knowledge of the anganwadi workers (AWWs) regarding growth monitoring (GM). A sound knowledge of the AWWs strengthens their skills and raises their capabilities to identify the children earliest moving towards malnutrition with the help of regular GM so as to take appropriate and early corrective action for further departure from good health. It also helps them as a teaching tool for empowering the mothers for preventive actions and better nutrition care of their children. Therefore, attempt has been made to discuss knowledge of AWWs about GM activities and the influence of reorientation training on their correct knowledge.

## Methodology

Since the inception of ICDS scheme, 28 ICDS projects are running in the urban slums as well as rural parts of the Delhi. For the study purpose, those districts were divided into four geographically demarcated zones - east, west, north and south. From each zone, one district was selected by simple random technique. In each district, five to eight ICDS projects are running. One project was selected from each zone by simple random selection technique. In each project, 90-176 anganwadi centers are functioning. Five AWCs are selected by systematic random selection technique. Thus, the total sample size was 20. All the AWWs from the selected AWCs were interviewed using semi-structured interview schedule to assess their knowledge regarding GM. The percent accuracy in the interpretation of growth chart and growth curve was analysed using spss version 10.1.

## Findings

### Profile of the AWWs

Eighty percent of AWWs were between 21 and 45 years of age. Thirty-five percent of AWWs were matriculate, fulfilling the criteria of educational qualification in their selection. Fifty percent of them were intermediates and 15% were even postgraduates. Ninety-five percent of them had working experience as AWW for more than 5 years and 70% were working at the present AWCs from the last 5 years [Table 1].

**Table 1:** Profile of the anganwadi workers

Variables	Number	Percentage
Age		
21-45 years	16	80
46-50 years	4	20
Educational qualification		
Matriculate	7	35
Intermediate	10	50
Postgraduate	3	15
Experience as anganwadi worker		
<5 years	1	5
5 years or more	19	95
Induction training of the anganwadi workers		
Yes	19	95
No	1	5
Reorientation training		
Yes	19	100
Frequency of reorientation training		
Once	6	32
Twice	8	42
Thrice	5	26

Except one AWW, all of them (95%) in the selected 20 centres had received 3 months induction training up to satisfactory level. Sixty-eight percent AWWs had received reorientation training for more than one time

### Knowledge of the AWWs

#### Mid upper arm circumference (MUAC) strips measurement:

Mid upper arm circumference strip has three colours - red, yellow and green. All the colours are meant to identify some grade of nutritional status of children. Regarding interpretation of the colours and action to be taken according to the colours, 50% of the AWWs of all the four zones (combined) had correct knowledge for the colours of MUAC strip as red for severe malnutrition, yellow for moderate malnutrition and green for no malnutrition; but only 35% AWWs were able to mention the correct action to be taken according to the interpreted colours of MUAC strip.

#### Growth chart:

Growth chart depicts the relationship between the age of the child and its weight from birth to 5 years of age. The graph depicts four curves - I, II, III and IV, representing 80, 70, 60 and 50% of the 50^th^ percentile of Harvard scale. As per the recommended norms, AWWs had to supply food to only those children who are Grade II or above malnutrition and children registered for preschool education. In the study area, it was found that 90% of the AWWs had the knowledge of growth chart interpretation but all of them were supplying food even to normal and grade I children.

#### Growth line interpretation:

Regular monitoring of weight of a child is plotted on the growth chart. Direction of the growth line (rising/flattened/falling) tells about the change in current nutritional status of a child. Seventy-five percent of the AWWs were able to interpret flattened growth line as no growth of a child and falling growth line as reduced nutritional status of the child, whereas, 80% of the AWWs were able to interpret raising growth line (for healthy children).

#### Major steps taken for growth monitoring:

Correct age estimation is essential for GM of a child. In addition, GM of malnourished children, irrespective of their age, is recorded monthly. Only 40% AWWs were able to mention that correct age estimation of the children is essential for GM and malnourished children should be weighed monthly.

## Discussion and Conclusion

Growth monitoring is one of the important activities conducted by the AWWs. For two-way communication with mothers, growth chart is the excellent tool. It not only clearly depicts the growth of a child to the mothers, but also gives early warning to both of them (AWWs and mothers) to take appropriate actions for children who are malnourished or going towards malnourishment.

In this study, most of the AWWs (13) had education qualification more than that prescribed for their selection. Nineteen AWWs had received induction training and most of them (13) had undergone reorientation training for more than one time. Majority of the AWWs were able to correctly interpret growth chart, and all of them were aware that children above 3 years should be followed up after every 3 months. But follow-up of malnourished children was known to only eight AWWs.

Through periodic recording of weight with respect to age, direction of growth curve provides more important information than the weight at one particular period, as downward dip or flat curve demands more specific attention on the nutrition of the child. Eighty percent of selected AWWs were able to interpret raising growth line for healthy child, while 15 AWWs (75%) mentioned falling growth line as reduced nutritional state of the child and flattened growth line as no weight gain by the child. Cross-tabulation of this aspect of the knowledge with frequencies of the training received had significant association, i.e. AWWs who had received reorientation training for more than one time had better knowledge [Table 2].

**Table 2:** Distribution of anganwadi workers' frequencies of reorientation training received in relation to their knowledge of growth monitoring

	Monthly weight record of malnourished children		Total
		
	Yes	No	
Frequency of reorientation training received			
Once	0	6	6
More than once	8	5	13
Total	8	11	19
Falling growth curve as reduced nutritional state			
Once	2	4	6
More than once	13	0	13
Total	15	4	19
Flattened growth curve as no weight gain by the child			
Once	2	4	6
More than once	13	0	13
Total	15	4	19

Chi-square test; *P* < 0.01

Certain minimum rules need to be followed for effective GM. For example, the AWW should estimate correct age of the child (in one month approximation) or malnourished children have to be given monthly follow-up irrespective of their age. In the selected AWWs of the four zones, only 40% AWWs knew that correct estimation of children's age is essential for GM or malnourished children should be weighed monthly irrespective of their age. Kapil *et al*.(5) had also mentioned that only 42% AWWs were able to mention the monthly weight recording of malnourished children. It was also found to have significant association with frequencies of the reorientation training received.

Thus, GM was devised as a tool for assessing the growth and development of a child, for detecting the earliest changes in the growth and to bring about appropriate response to ensure that growth continues uninterrupted. Flattening of the growth curve is the earliest sign of protein energy malnutrition and may precede clinical sign by weeks or even months. The whole purpose of GM is to detect growth flattening.

Repeated practical reorientation training has significant impact to strengthen the correct knowledge of the AWWs, which increases their capabilities to take corrective and preventive action at appropriate time for optimum development of the children.
